# Ondex Web: web-based visualization and exploration of heterogeneous biological networks

**DOI:** 10.1093/bioinformatics/btt740

**Published:** 2013-12-20

**Authors:** Jan Taubert, Keywan Hassani-Pak, Nathalie Castells-Brooke, Christopher J. Rawlings

**Affiliations:** Rothamsted Research, Computational and Systems Biology, Harpenden, AL5 2JQ, UK

## Abstract

**Summary:** Ondex Web is a new web-based implementation of the network visualization and exploration tools from the Ondex data integration platform. New features such as context-sensitive menus and annotation tools provide users with intuitive ways to explore and manipulate the appearance of heterogeneous biological networks. Ondex Web is open source, written in Java and can be easily embedded into Web sites as an applet. Ondex Web supports loading data from a variety of network formats, such as XGMML, NWB, Pajek and OXL.

**Availability and implementation:** http://ondex.rothamsted.ac.uk/OndexWeb.

**Contact:** keywan.hassani-pak@rothamsted.ac.uk

## 1 INTRODUCTION

The integrative approach to systems biology studies the interactions between molecular or cellular components to understand how a behavior or phenotype might emerge from the properties or structure of the interactions. This approach generally requires data from diverse sources to be integrated and analyzed together.

It is common in systems biology to adopt network formalisms to represent, visualize and explore interaction information, and a number of web-based applications are available for the visualization of biological networks (Gehlenborg *et al.*, 2010). A well-known example is Cytoscape Web (Lopes *et al.*, 2010); version 1 of which is implemented using Flex/ActionScript and a JavaScript API. Cytoscape Web can visualize small- to medium-sized networks, (i.e. a few hundred nodes and edges). The visual styles (e.g. color, size and opacity) of nodes and edges can be dynamically changed by the client. However, loading of data and customization of its appearance in Cytoscape Web require extensive JavaScript programming skills.

**Fig. 1.**
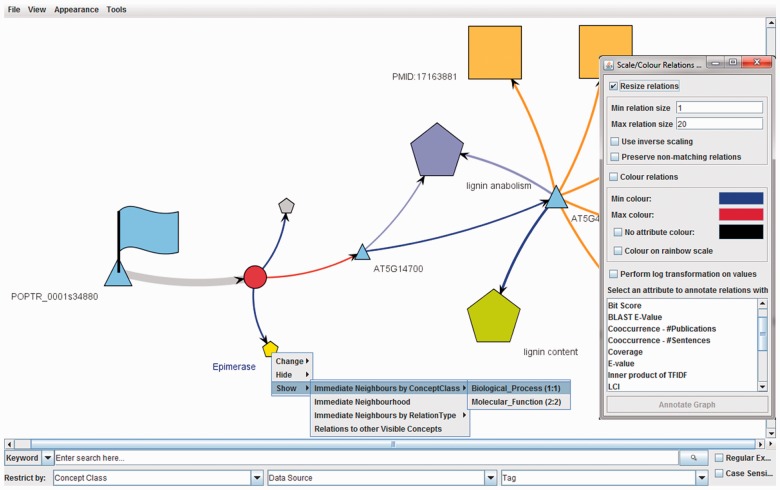
A heterogeneous biological network is displayed in Ondex Web. Right-click on nodes and edges to bring up a context-sensitive menu. Annotators change the appearance of the network based on node/edge properties. The search menu allows searching loaded data and across web services

We have previously developed the Ondex software suite (Köhler *et al.*, 2006) (www.ondex.org) to provide a general framework for the integration of heterogeneous biological data. We describe here a new implementation of the visualization software from the Ondex suite that enables integrated networks and associated quantitative data to be presented in a web browser. This enables delivery of results from pre-integrated datasets to end-users, as featured in the QTLNetMiner system (http://ondex.rothamsted.ac.uk/QTLNetMiner/), and overcomes the significant obstacle encountered by many biologists of installing and learning complex data integration software system. Ondex Web has been built as a Java applet, reusing proven functions and plugins from the Ondex desktop application. This results in a richer repertoire of functions for exploring and manipulating heterogeneous information networks (Sun and Han, 2012) than currently available in Cytoscape Web. Ondex Web provides new features not described before, including easy-to-use search functions for loaded data and across web services (e.g. ChEMBL, UniProtKB). The network appearance can be changed using dedicated plugins, and another new feature is the extensive context-sensitive menus on nodes and edges (Fig. 1).

Ondex Web has direct support for loading data in common network formats such as Cytoscape’s XGMML (Punin and Krishnamoorthy, 2001), NWB (NWB, 2006), Pajek (Batagelj and Mrvar, 2002). The Ondex desktop application can be used to prepare datasets integrated from a large range of input formats (see http://ondex.org/formats.html) and exported in OXL format (Taubert *et al.*, 2007) for use in Ondex Web. Example data sets are available for download (http://ondex.org/doc.html).

## 2 FEATURES

Ondex Web supports two complementary strategies for the exploration and analysis of biological networks. The bottom-up strategy, which is recommended for very large integrated datasets, starts with a few nodes (e.g. from a search query). The new context-sensitive right-click menus on nodes and edges can be used to iteratively build a larger network by exploration. The top-down strategy, used for small- or medium-sized networks, displays the entire network, and parts of the network can be hidden using right-click context menus or highlighted using annotation tools.

### 2.1 Exploring heterogeneous biological networks

A heterogeneous information network consists of multi-typed objects (e.g. gene, pathway, ontology, literature) and semantic links (e.g. encodes, interacts, expressed, published). This semantic information is used to automatically map visual attributes like node shape, node color or edge color.

Starting with a small part of the loaded network displayed, a user can, for example, follow a bottom-up strategy by right-clicking on a particular node or selection of nodes and then using the context-sensitive menu to change the visibility property of neighboring nodes. The context-sensitive menus ensure that only valid operations are presented to the user. For example, if a gene does not have any known proteins, then the menu item for showing encoded proteins would not appear.

If additional information about the originating data source of nodes and edges has been recorded as a URL, then a hyperlink to the corresponding web page is presented at the top of the context menu, enabling the user to easily obtain more information from the web. The complete list of information on a node or edge in the network is accessible using the “Item Info” (“View” menu) function of Ondex Web.

### 2.2 Advanced search

Ondex Web offers a new advanced search function, which can be shown via the “View” menu. The user can enter any keyword or regular expression to search all the information held in the loaded network. The search can be restricted to nodes of a given concept class or data source. It is also possible to search external web services like ChEMBL and UniProtKB using corresponding IDs, which will add search results as new nodes to the network. The web-service interface can also be used to run server-side data analysis tasks, as also exploited in the QTLNetMiner application.

The search results are displayed in a separate frame in a tabular format. The matching text is highlighted. Selecting one or more rows in the table propagates to the selection of the relevant nodes in the network, immediately highlighting the search results in the visualization. Furthermore, the network can be filtered down to a specified neighborhood surrounding the search results.

Once the user has obtained the most informative network for their problem using the methods outlined above, it is possible to either save the network in OXL format, or the current view can be exported as an image (JPG, BMP, PNG) or vector graphics (EPS).

## 3 IMPLEMENTATION

Ondex Web was refactored from the Java libraries used in the Ondex desktop application. This ensures consistent behavior for users of both tools. Ondex Web requires Oracle Java version 6 or higher on the client side. Once loaded, the applet is cached by Java, which makes subsequent calls much faster. The user can set the size of the client-side applet memory. The applet HTML tag of Ondex Web provides some parameters for customizing the initial look and interaction with the displayed network.

### 3.1 Performance and availability

Ondex Web runs as a Java client applet in a web page, and is therefore only limited by available client resources and the Java runtime environment. In particular the more memory available to the Java virtual machine on the client, the larger networks can be loaded. The pragmatic limitations of visualizing and interacting with large networks led us to provide a warning to the user if a larger than usual network is to be displayed. The user can then choose to adapt their strategy to a bottom-up style or change from default initial force-directed layout to a faster, but simpler, initial one.

The Ondex Web application and source code is available from http://sourceforge.net/projects/ondex/files/OndexWeb/.

## 4 CONCLUSION

Heterogeneous biological networks require good exploration and visualization tools. Ondex Web is free, open-source and can easily be embedded on Web sites to visualize networks. It comes with a rich and intuitive set of functions to explore and manipulate biological networks. For an example application of Ondex Web, visit QTLNetMiner (http://ondex.rothamsted.ac.uk/QTLNetMiner/), where it is used to visualize evidence networks supporting candidate gene selections.

*Funding*: Rothamsted Research receives grant in aid from the Biotechnology and Biological Sciences Research Council (BBSRC). BBSRC SABR award (BB/F006039/1) and TSB project (TP 5082-33372).

*Conflict of Interest*: none declared.
